# Effects of essential oils on calf growth, ruminal fermentation, and antioxidative status: a meta-analysis

**DOI:** 10.3389/fvets.2025.1573846

**Published:** 2025-06-02

**Authors:** Wei Li, Fang Wang, Yongsheng Han, Fang Sun, Chunhai Liu, Yuanfang Zhu, Peng Zhong

**Affiliations:** ^1^Heilongjiang Academy of Agricultural Sciences Livestock Veterinary Branch, Qiqihar, China; ^2^Heilongjiang Provincial Key Laboratory of Resistance Gene Engineering and Protection of Biodiversity in Cold Areas, College of Life Sciences and Agroforestry, Qiqihar University, Qiqihar, China; ^3^Liaoning Feidi Feeding Technology Co., Ltd, Xingcheng, China

**Keywords:** meta-analysis, essential oil, calves, ruminal fermentation, anti-oxidative status

## Abstract

**Introduction:**

Essential oils (EO) have gained significant attention in the calves industry due to their antimicrobial properties. This meta-analysis aimed to evaluate the efficacy of EO on calves to provide better guidance for cattle feed.

**Methods:**

We conducted a comprehensive search of relevant studies published from inception to February 6, 2022, using PubMed, CENTRAL,Web of Science, and EMBASE. The quality of included studies was assessed using the CAMARADES checklist. Effect sizes were calculated using weighted mean differences (WMD) for continuous variables and summary risk ratios (RR) for binary variables. Subgroup and sensitivity analyses were also performed.

**Results:**

This systematic review and meta-analysis included 10 animal studies with 226 calves. The average quality score was 5.8 (range: 5–7). EO improved milk production (WMD = 0.30; 95% CI 0.13 to 0.47; *I^2^* = 0%, *p* = 0.985) and beta-hydroxybutyric acid levels (WMD = 0.01; 95% CI 0.04 to 0.16; *I^2^* = 0%, *p* = 0.472). However, EO did not significantly improve rumen fermentation characteristics or overall performance index.

**Discussion:**

While EO may have beneficial effects on specific outcomes like milk production and beta-hydroxybutyric acid levels,its impact on rumen fermentation and overall performance remains inconclusive. Future large-scale randomized controlled trials (RCTs) are needed to better assess the effects of EO on ruminal fermentation efficiency, anti-oxidative status, and overall performance.

## Introduction

In the feed diets of beef cattle, cereal grains are usually used to increase performance and feed efficacy. However, highly fermentable substances in these diets can decrease ruminal pH and increase the risk of acidosis and bloat (1). Therefore, antibiotics have been successfully used in beef diets to improve nutrient utilization efficiency and reduce the incidence of ruminal acidosis and bloat. Nevertheless, the use of antibiotics often results in residues in milk and meat, which can affect human health (2). As public demand for reduced use of additives in animal feed diets, the European Union proposed regulations to ban antimicrobials in 2003. This has put enormous pressure on scientists and health authorities to reduce the use of antibiotics in feed production. Effective alternatives to antimicrobials are receiving increasing attention (3–5).

Essential oils (EO) are naturally occurring minor component metabolites and volatile components extracted from plants by distillation methods, primarily by steam distillation (6). Chemically, EO is a complex mixture of monoterpenes and sesquiterpenes and biologically relevant phenols or monophenols (7). EO has antimicrobial activities against gram-negative and gram-positive bacteria. The antimicrobial properties of essential oils regulate rumen fermentation (8). EO, for example, oregano oil containing high concentrations of phenolic compounds, was evaluated as a modulator of rumen fermentation (9). EO has attracted much attention due to its favorable antimicrobial properties as an alternative to commonly used antimicrobial agents in livestock production (10, 11).

Some *in vivo* studies have identified the efficacy of EO. In 2001, Landete-Castillejos et al. (9) observed that EO increased total volatile fatty acids (VFA) concentration in 24 h batch fermentation, which energetically benefits the ruminant animal. In 2013, Vakili et al. (12) reported an increase in the molar proportion of propionate in ruminal fluid collected from beef cattle-fed diets supplemented with EO. Several short-term *in vitro* studies have shown that EO affects N metabolism via the reduction of protein degradation and ammonia production (13, 14). In 2008, Macheboeuf et al. (15) observed that EO decreased methane production (up to a 98% decrease). In the same study, the authors reported lower anti-methanogenic activity of carvacrol, suggesting that other components present in lower concentrations in EO may have acted antagonistically with carvacrol, thereby attenuating the anti-methanogenic properties of EO.

Due to the limitations of the *in vitro* technique (i.e., short-term culture, buffered media, and inability to replicate the diversity and viability of rumen microbial populations), the data should be interpreted cautiously. High-quality meta-analysis has always been regarded as the best evidence and provides credible suggestions. Therefore, this systematic review and meta-analysis aimed to determine the efficacy and safety of essential oils on ruminal fermentation, anti-oxidative status, and calf performance as comprehensively as possible and to provide better guidance for cattle feed.

## Methods and method

This study was reported in line with Preferred Reporting Items for Systematic Reviews and Meta-Analyses(PRISMA).

### Data sources and searches

CENTAL, Embase, PubMed, and Web of Science were searched from the earliest publication date to February 06, 2023. We also screened the reference lists of relevant reviews. The search terms included related text words and medical subject headings regarding “calf” and “essential oil.” We tailored search strategies for each database. Details of the search strategies were provided in Supplementary Table S1.

### Study selection

Two independent reviewers screened titles, abstracts, and full texts and agreed on the final included studies. When disagreements arose, a third investigator was consulted. Studies were considered eligible if they (1) included cow or calf, (2) included the intervention group using essential oils as feed additives, (3) included the control group using no additive feed, (4) the research results need to include the effect of essential oil addition on rumen fermentation. There are no restrictions on the research design. Non-English literature, *in vitro* studies, single-arm studies, studies without full-text and statistical methods, and literature with the repeated publication of research results were excluded.

### Justification for inclusion and exclusion criteria

The inclusion and exclusion criteria were designed to ensure the studies included were relevant and of sufficient quality to provide meaningful insights. The criteria were as follows: Inclusion criteria: Studies were included if they (1) included cow or calf, (2) included the intervention group using essential oils as feed additives, (3) included the control group using no additive feed, and (4) reported the effect of essential oil addition on rumen fermentation. Exclusion criteria: Studies were excluded if they were (1) *in vitro* studies, (2) single-arm studies without a control group, (3) lacked full-text availability, (4) had incomplete statistical data, or (5) were duplicate publications.

Given the involvement of live animals in the studies included in this meta-analysis, it is crucial to address the ethical considerations and regulatory compliance related to animal experimentation. All studies included in this meta-analysis were conducted in accordance with the ethical guidelines for the use of animals in research, which emphasize the principles of Replacement, Reduction, and Refinement (the 3Rs). These guidelines aim to minimize the use of animals and ensure their well-being throughout the experimental procedures.

### Data extraction and quality assessment

Reviewers independently extracted data in a standardized form. The following data were extracted: (1) general information of the included studies, including the first author, published year, type of intervention, composition, duration, and dose of intervention, and baseline characteristics of the calf; (2) rumen fermentation characteristics, including PH, NH3-N (mg/dL), Total VFA (mM), Butyrate, Acetate: propionate, ammonia (mg/dL), Protozoa. (3) blood metabolites, including Urea N (mg/dl), beta hydroxyl butyric acid (mM), and Glucose (mg/dL). (4) performance, including Body weight (kg), Withers height (cm), and Heart girth (cm). (5) Feed efficiency (milk/dry matter intake), Milk production (kg/d). Two authors independently assessed the risk of bias. Any disagreements were resolved via discussion among the authors. The quality of included studies was evaluated using the “Collaborative Approach to Meta-Analysis and Review of Animal Data from Experimental Studies” (CAMARADES) checklist with ten items. The CAMARADES checklist is used to perform a combined assessment of the reporting of several measures to reduce bias and several indicators of external validity and study quality.

### Data synthesis and analysis

STATA Version 14.1 (Stata Corporation, College Station, Texas, USA) was used to calculate the weighted mean difference (WMD) as the effect size for the continuous variables. Summary RR with 95% confidence intervals (CI) were presented if the results were binary variables. Using a fixed effects model, the summary RR and 95% CIs were calculated. The random effects model was used if high heterogeneity. The extent of heterogeneity was interpreted by the total percentage of variation between the studies concerned, measured by the *I*^2^ statistic. The *I*^2^ value was categorized as low if *I*^2^ was 0%e25%, moderate if *I*^2^ was 25%e50%, and high if *I*^2^ was >50%. Additionally, Q-statistic was used to assess the presence of heterogeneity. P statistic 0.05 was considered to indicate no significant heterogeneity among the included studies. Subgroup analysis was conducted according to the different subtypes of interventions.

The publication bias test would not be necessary to analyze if the number of included trials was less than ten.

## Results

### Search results

**Figure 1:**
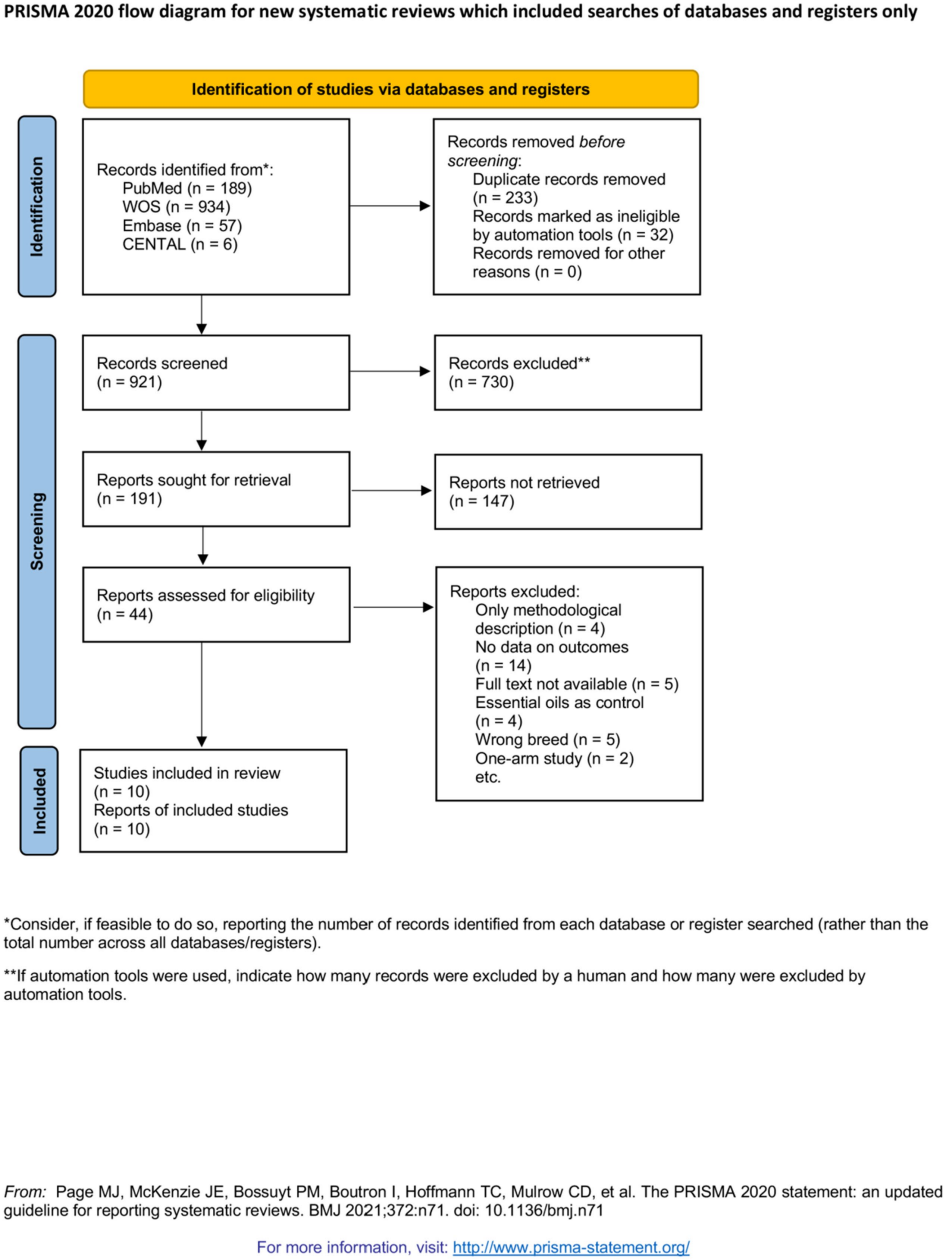
PRISMA 2020 flow diagram for new systematic reviews which included searches of databases and registers only.

A total of 1,186 publications were identified, and 995 publications were excluded. Among them, 265 publications were excluded for duplication, and 730 were excluded after screening titles and abstracts. Ultimately, 44 full-text publications were assessed for eligibility. Of the 44 retrieved publications, 34 were excluded, and five studies were identified as eligible for inclusion in this review (Figure 1).

### Study characteristics

The key characteristics of all included studies were summarized in Table 1. All included studies were published from 2013 to 2021. A total of 226 calves were included in this meta-analysis, 142 in EO intervention groups and 84 in placebo control groups. The interventions varied in their dose and intervention periods. The duration of the intervention ranged from 21 days to 115 days.

**Table 1:** The characteristics of included studies.

Author	Year	Control group			Intervention group			Period (day)
		Control	Dose	Sample size	Intervention	Dose	Sample size	
M. Akbarian-Tefaghi	2017	without essential oils	NA	11	Phytogenic feed additive containing EO	3 g/kg /day	11	67
Olga Teresa Barreto Cruz	2014	without essential oils	NA	10	EO from cashew and castor	3 g/animal/day	10	115
C. Benchaar	2019	without essential oils	NA	2	Oregano oil	50 mg/kg	2	26
C. Benchaar	2020	without essential oils	NA	2	Oregano oil	50 mg/kg	2	28
C. Benchaar	2006	without essential oils	NA	2	Oregano oil	50 mg/kg	2	28
Joana Palhares Campolina	2021	without essential oils	NA	14	Commercial blend of EO	1 g/day/calf	29	90
S. N. S. e Silva	2021	without essential oils	NA	2	Natural EO with carvacrol, cinnamaldehyde, and limonene	16 g/cow/day	8	21
Matteo Mezzetti	2021	without essential oils	NA	18	EO	50 g/cow/day	36	35
F. H. R. Santos	2015	without essential oils	NA	15	EO with milk replacer	400 mg/kg	30	70
A. R. Vakili, B. Khorrami	2013	without essential oils	NA	8	basal diet supplemented with thyme oil	5 g/calf daily	12	45

This table provides detailed information on the included studies, including the first author, publication year, intervention details, sample size, and intervention period. Statistical significance is indicated where applicable.

### Risk of bias in included studies

The average was 5.8, ranging from 5 to 7. Three studies were rated as five scores, six studies were rated as six scores, and only one study was rated as seven scores. No studies reported allocation concealment, blinded assessment of outcome, use of animals with cancer, sample size calculation, statement of compliance with regulatory requirements, physiological monitoring, and reporting animals excluded from the analysis. The results were shown in Table 2.

**Table 2:** The quality assessment of included studies.

Items	1	2	3	4	5	6	7	8	9	10
Publication in peer-reviewed journal	1	1	1	1	1	1	1	1	1	1
Statement of control of temperature	1	0	1	1	1	1	1	0	0	1
Randomization to treatment and control	1	1	1	1	1	1	1	1	1	1
Allocation concealment	0	0	0	0	0	0	0	0	0	0
Blinded assessment of outcome	0	0	0	0	0	0	0	0	0	0
Avoidance of intrinsically neuroprotective aesthetics	1	0	0	0	0	0	0	0	0	0
Use of animals with cancer	0	0	0	0	0	0	0	0	0	0
Sample size calculation	0	0	0	0	0	0	0	0	0	0
Statement of compliance with regulatory requirements	0	0	0	0	0	0	0	0	0	0
Statement regarding possible conflict of interest	1	1	1	1	1	1	1	1	1	1
Physiological monitoring	0	0	0	0	0	0	0	0	0	0
Prespecified inclusion and exclusion criteria	1	1	1	1	1	1	1	1	1	1
Reporting animals excluded from analysis	0	0	0	0	0	0	0	0	0	0
Reporting of study funding	1	1	1	1	1	1	1	1	1	1
Total score	7	5	6	6	6	6	6	5	5	6

This table evaluates the quality of included studies using the CAMARADES checklist. Each item is scored, and the total score is provided. Statistical significance is indicated where applicable.

### Rumen fermentation characteristics

**Figure 2:**
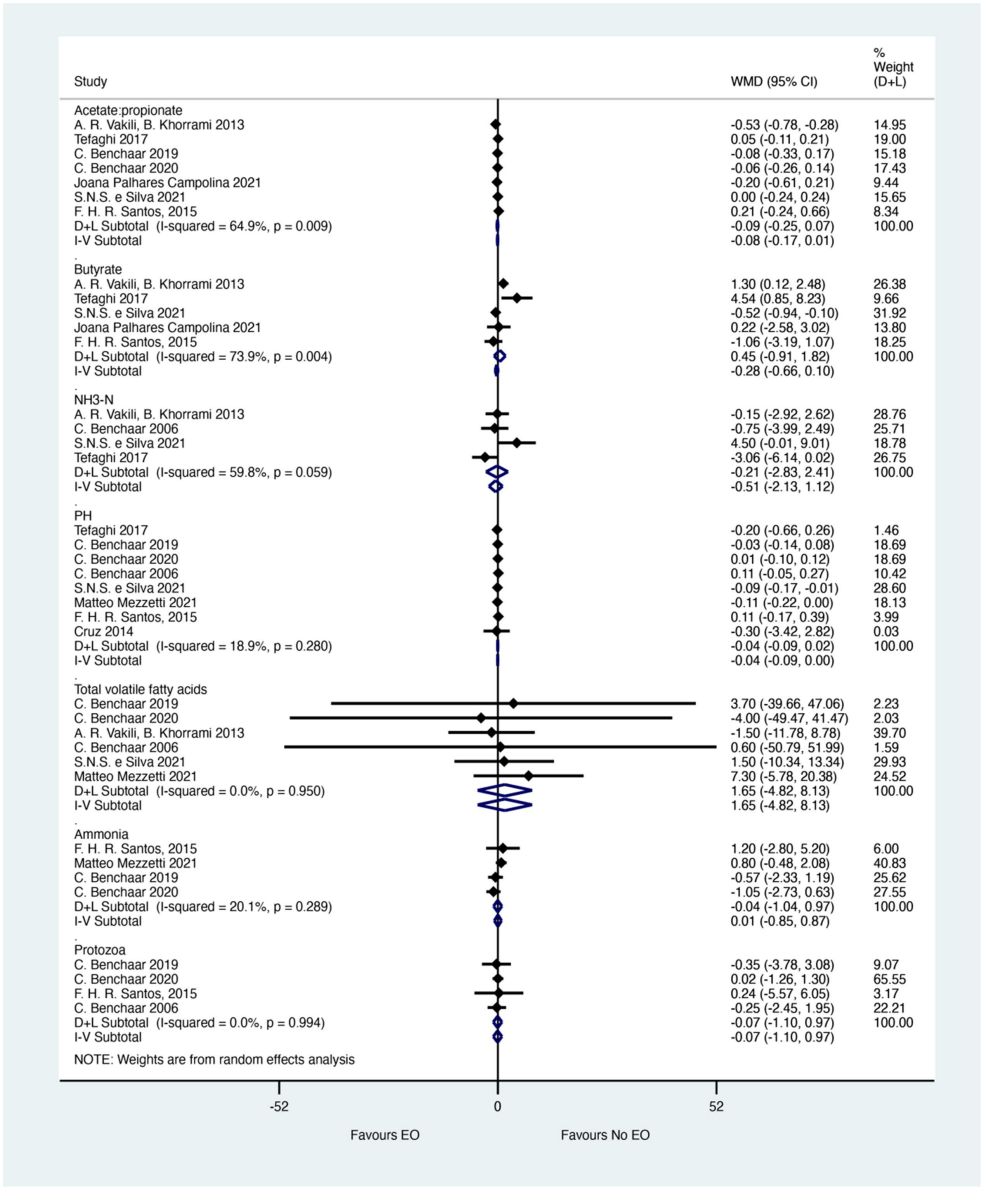
Merged effects of essential oils on rumen fermentation characteristics. The figure shows the weighted mean differences (WMD) and 95% confidence intervals (CI) for various rumen fermentation parameters. The effect sizes indicate the magnitude of change in each parameter due to essential oil supplementation. Asterisks (*) denote statistical significance (*p* < 0.05).

The merged effects of Rumen fermentation characteristics were shown in Figure 2. Seven studies reported Acetate: propionate ratios. No significant difference was observed between EO and control groups (WMD = −0.08; 95% CI −0.17 to 0.01; *I^2^* = 64.9%, *p* = 0.009). However, the effect size (WMD) of −0.08 indicates a small but potentially meaningful difference in the ratio of Acetate to propionate, which could suggest a slight shift in rumen fermentation pathways. This effect size, although not statistically significant, may still be biologically relevant in certain contexts and warrants further investigation. Butyrate levels were analyzed in seven studies. The meta-analysis showed no significant difference between EO and control groups (WMD = −0.28; 95% CI −0.66 to 0.10; *I^2^* = 73.9%, *p* = 0.004). The effect size (WMD) of −0.28 suggests a moderate decrease in butyrate levels in EO-treated calves, which could have implications for rumen health and energy metabolism. However, the wide confidence interval and high heterogeneity (*I^2^* = 73.9%) indicate that this effect is not consistent across studies and requires further exploration. Four studies involving 103 calves reported NH3-N. Similarly, no significant difference between EO and control groups (WMD = −0.51; 95% CI −2.13 to 1.12; *I^2^* = 59.8%, *p* = 0.059) were shown in Figure 2. The effect size (WMD) of −0.51 indicates a small reduction in NH3-N levels, which could be beneficial for reducing ammonia emissions and improving air quality in calf housing. However, the wide confidence interval and moderate heterogeneity (*I^2^* = 59.8%) suggest that this effect is not robust and needs to be confirmed in larger studies.

### Blood metabolites

**Figure 3:**
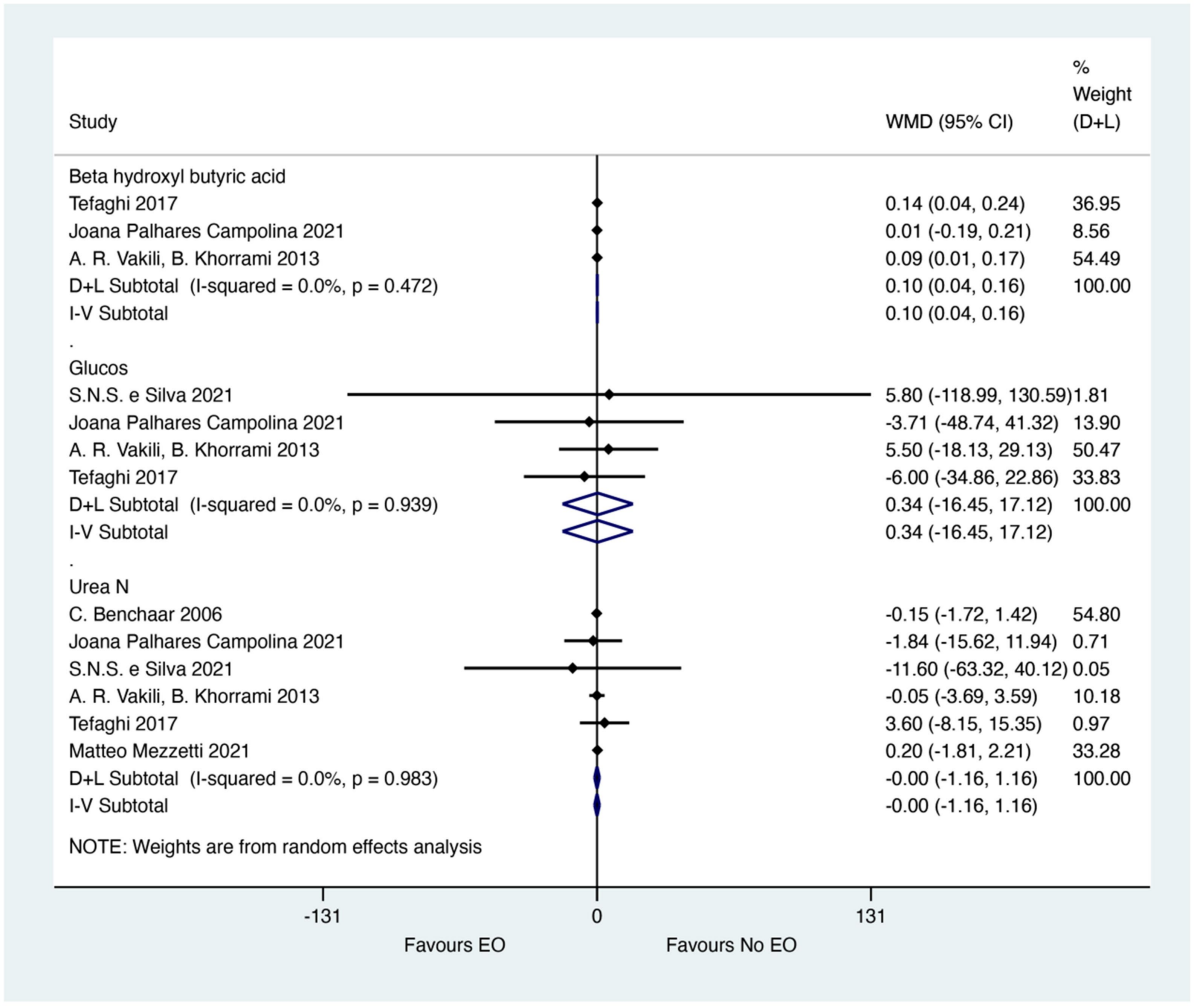
Meta-analysis results for blood metabolites. The figure displays the weighted mean differences (WMD) and 95% confidence intervals (CI) for key blood metabolites, including beta-hydroxybutyric acid, glucose, and urea nitrogen. Asterisks (*) indicate statistically significant differences (*p* < 0.05).

Figure 3 showed the meta-analysis results for blood metabolites. EO could significantly improve the Beta hydroxyl butyric acid (WMD = 0.01; 95% CI 0.04 to 0.16; *I^2^* = 0%, *p* = 0.472). The effect size (WMD) of 0.01 indicates a small but statistically significant increase in beta-hydroxybutyric acid levels, which could be biologically relevant for improving energy metabolism and reducing the risk of ketosis in calves. This effect size, although small, suggests a potential benefit of EO supplementation in enhancing metabolic health. For Glucose and Urea N, there was no significant difference between EO and control groups (WMD = 0.34; 95% CI −16.45 to 17.12; I^2^ = 18.9%, *p* = 0.939), (WMD = 0.00; 95% CI −1.16 to 1.16; I^2^ = 0%, *p* = 0.983). The effect sizes (WMD) of 0.34 for Glucose and 0.00 for Urea *N* indicate no meaningful differences in these blood metabolites, suggesting that EO supplementation does not significantly impact glucose metabolism or nitrogen balance in calves.

### Performance

**Figure 4:**
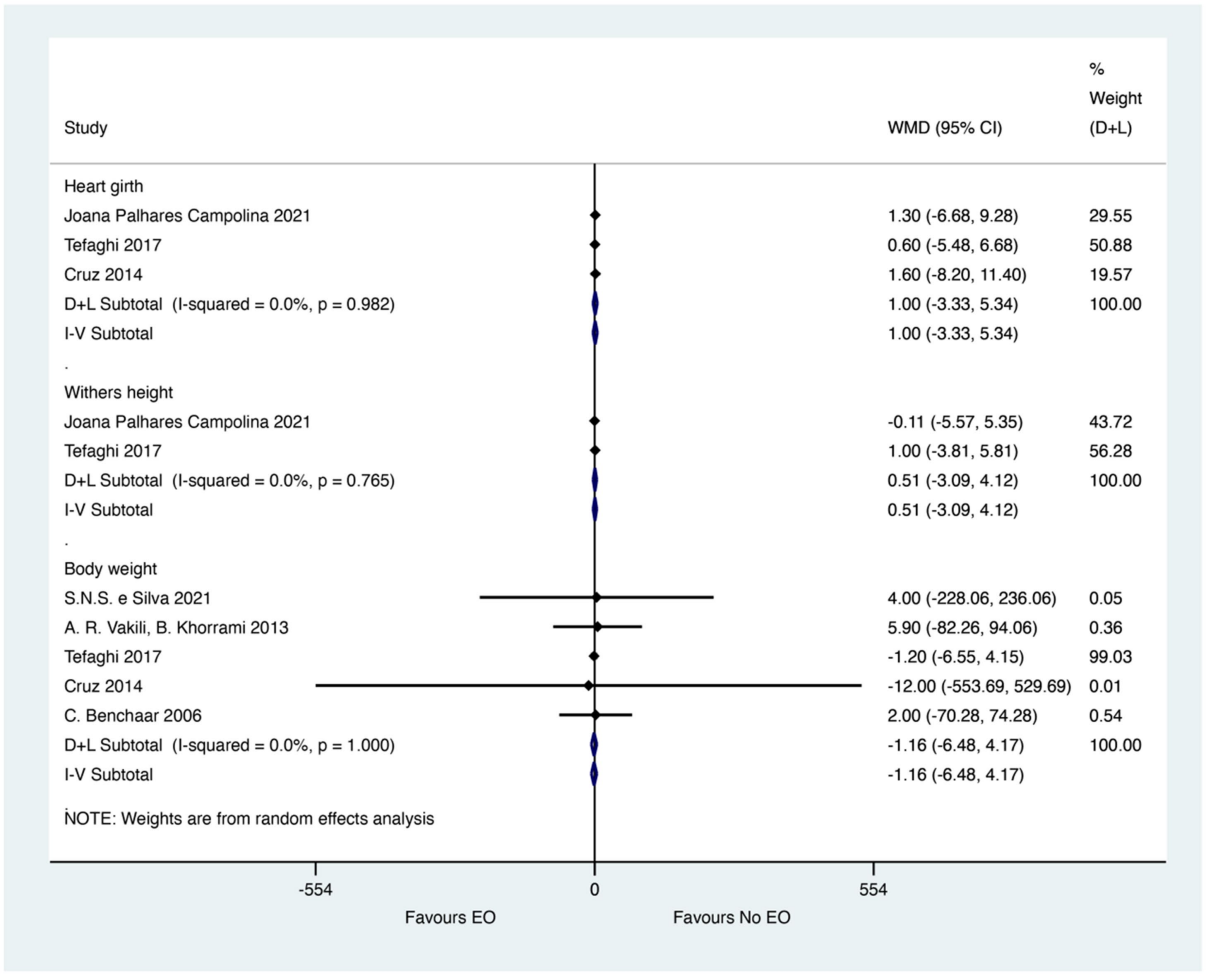
Merged effects of essential oils on calf performance. The figure presents the weighted mean differences (WMD) and 95% confidence intervals (CI) for performance indicators such as heart girth, withers height, and body weight. Asterisks (*) denote statistical significance (*p* < 0.05).

The merged effect for the performance of calves was presented in Figure 4. EO could not significantly improve the heart girth from three studies (WMD = 1; 95% CI −3.33 to 5.34; *I^2^* = 0%, *p* = 0.982). The effect size (WMD) of 1 indicates no meaningful difference in heart girth, suggesting that EO supplementation does not significantly impact this measure of calf growth. Besides, the data from two and five studies showed that EO could not significantly improve the Withers height (WMD = 0.51; 95% CI −3.09 to 4.12; *I^2^* = 0%, *p* = 0.765) and Body weight (WMD = −1.16; 95% CI −6.48 to4.17; *I^2^* = 0%, *p* = 0.100). The effect sizes (WMD) of 0.51 for Withers height and −1.16 for Body weight indicate no meaningful differences in these measures of calf performance, suggesting that EO supplementation does not significantly impact growth and development in calves.

### Feed efficiency

**Figure 5:**
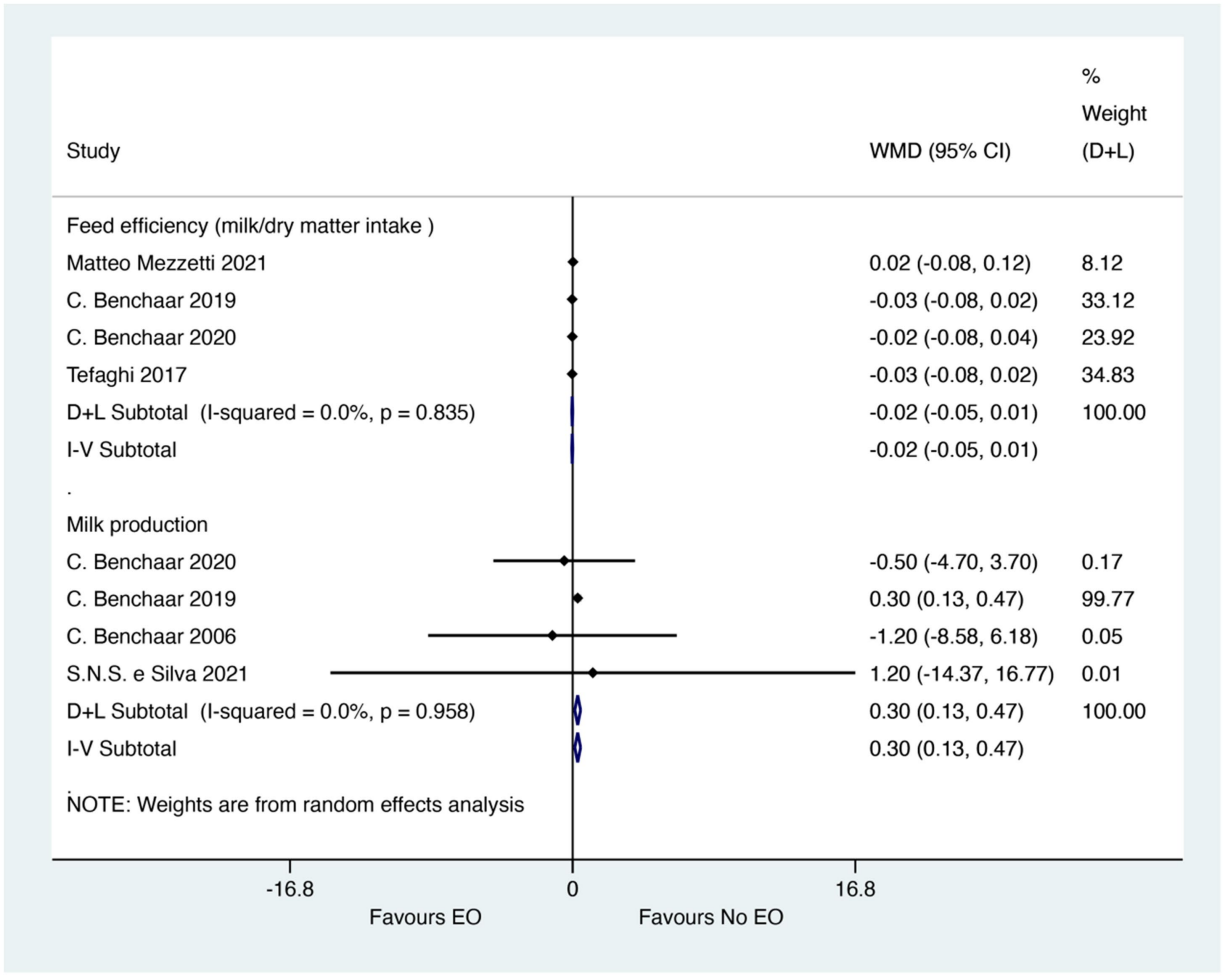
Forest plot of essential oils on feed efficiency and milk production. The figure shows the weighted mean differences (WMD) and 95% confidence intervals (CI) for milk production and milk/dry matter intake. Asterisks (*) indicate statistically significant differences (*p* < 0.05).

The meta-analysis showed that EO could not significantly improve milk production (WMD = 0.30; 95% CI 0.13 to 0.47; *I^2^* = 0%, *p* = 0.985), but EO did not have efficacy on milk/dry matter intake (WMD = −0.02; 95% CI −0.05 to 0.01; *I^2^* = 0%, *p* = 0.835). The effect size (WMD) of 0.30 for milk production indicates a small but statistically significant increase in milk production, which could be biologically relevant for improving feed efficiency and economic returns in calf rearing. However, the lack of significant effect on milk/dry matter intake suggests that this improvement in milk production may not be directly related to increased feed intake but could be due to other factors such as improved metabolic efficiency. The forest plot was shown in Figure 5.

### Publication bias

**Figure 6:**
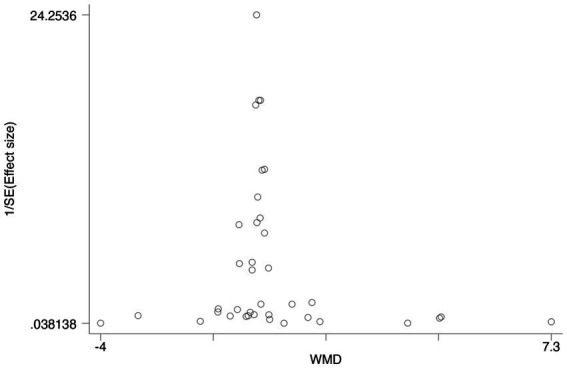
The funnel plot of the OR for publication bias.

The publication bias test was unnecessary since the included trials were less than ten in analyzed outcomes. The funnel plot of the OR for publication bias suggested the absence of bias because of plot symmetry (Figure 6).

## Discussion

EO have a variety of probiotic benefits in animal husbandry, including anti-inflammatory, antioxidant and *in vitro* deworming (16–18). EO may have antimicrobial properties, potentially representing a methane mitigation strategy suitable for organic production (19–21). EO has a complex mix of many compounds and has a major role in an antimicrobial activity tested for its effect on rumen fermentation using a batch culture technique (22, 23). EO has antimicrobial activity against a broad spectrum of Gram-positive and Gram-negative bacteria, and its potential effect on modifying rumen microbial fermentation has been recently studied (24–26). In this systematic review and meta-analysis of 10 animal studies, including 226 calves, EO could improve milk production and beta hydroxyl butyric acid. However, EO did not significantly improve rumen fermentation characteristics and performance index.

In small ruminants, several studies have evaluated the effect of dietary inclusion of EOs on animal performance, nutrient digestibility, ruminal fermentation, blood biochemistry, meat quality, and milk production and composition (27–29). According to the meta-analysis conducted by Dorantes-Iturbide et al., EOs could improve the taste and palatability of livestock foods with beef cattle (30). Besides, various EOs have been shown to increase the abundance of fungi and ruminal bacteria associated with fiber degradation in the rumen (21, 31, 32). In a meat analysis for six kinds of small ruminants, EOs were found to increase dry matter intake (6). Consistent with these findings, our results show that EO improved milk production, but did notsignificantly affect milk/dry matter intake, possibly due to the indirect role of dry matter intake (33).

Butyric acid is a short-chain C2–5 organic fatty acids (34), is the primary end-product of anaerobic bacterial carbohydrate fermentation in the rumen of certain bovine species. *In vivo* studies have shown that adding Butyric acid to acidified milk could affect gastrointestinal tract metabolism and development in calves (7). Butyric acid supplements can improve small intestine development in calves, reducing diarrhea rate and alleviating weaning stress (35, 36). In our study, EO improve beta-hydroxyl butyric acid, which may have indirect efficacy on digestibility with better intestinal development for calves.

Although most of the included studies were not RCTs and lacked high methodology quality, they exhibited similar biases and limitations to those in our current study (22, 37). The small size may have weakened the strength of the evidence. Additionally, the varying intervention periods and observations could have acted as potential confounding factors in assessing efficacy. For most outcomes, EO did not significantly alter performance or rumen fermentation characteristics (38, 39). However, these previous studies primarily focused on the synergistic effects of EO and other nutrient intakes. A larger-scale trial would provide a better assessment of EO’s impact on milk production efficiency.

Compared to no EO addition, supplementing cattle feed with essential oils demonstrated positive effects, primarily through impacts on the gastrointestinal tract (GIT). EO supplementation increased digestibility, improved pancreatic enzyme activity, changed microbiota, affected amino acid absorption in the intestines, and consequently, improved feed conversion rates (39–41). In our study, EO improved milk production and beta-hydroxyl butyric acid levels. EOs represent a viable health additive option for modern production systems and can serve as an alternative to improve calf health and performance.

In summary, EO supplementation showed potential benefits in improving milk production and beta-hydroxybutyric acid levels in calves. However, its effects on rumen fermentation and overall performance were not significant. Further large-scale RCTs are needed to comprehensively evaluate the efficacy of EO in enhancing ruminal fermentation, anti-oxidative status, and performance in calves.

## Data Availability

The original contributions presented in the study are included in the article/Supplementary material, further inquiries can be directed to the corresponding author.
