# Supplementary material for: Effects of essential oils on calf growth, ruminal fermentation, and antioxidative status: a meta-analysis
Source: Front Vet Sci. 2025 Jun 2;12:1573846. doi: 10.3389/fvets.2025.1573846 (PMC12171130; doi:10.3389/fvets.2025.1573846)
Supplement: Supplementary file 1 [file Table_1.DOCX]

Table S1 the search strategy

| **Database** | **Strategy and results** |
| --- | --- |
| CENTAL | #1 'calf' 2987  #2 'cattle' OR 'dairy cow' 2359  #3 'oils, volatile' OR 'volatile oils' OR ('essential' AND 'oils') OR 'essential oils' 1360  #4 #1 OR #2 5142  #5 #3 AND #4 11  #6 'digest' OR 'digestability' OR 'digestable' OR 'digestate' OR 'digestates' OR 'digested' OR 'digester' OR 'digesters' OR 'digestibilities' OR 'digestibility' OR 'digestible' OR 'digesting' OR 'digestion' OR 'digestions' OR 'digestive system'OR 'digestive system' OR 'digestive' OR 'digestives' OR 'digests' 22151  #7 'carcass' OR 'carcasses' 229  #8 ('ruminal' OR 'ruminally' OR 'ruminant s' OR 'ruminants' OR 'ruminant') AND ('ferment' OR 'fermentabilities' OR 'fermentability' OR 'fermentable' OR 'fermentate' OR 'fermentated' OR 'fermentates' OR 'fermentation' OR 'fermentations' OR 'fermentative' OR 'fermentatively' OR 'fermentator' OR 'fermented' OR 'fermenter' OR 'fermenters' OR 'fermenting' OR 'fermention' OR 'ferments') 105  #9 ('blood' OR 'bloods' OR 'haematology' OR 'hematology' OR 'haematoma' OR 'hematoma' OR 'haemorrhage' OR 'hemorrhage' OR 'haemorrhages' OR 'hemorrhages' OR 'haemorrhagic' OR 'haemorrhaging' OR 'hematologies' OR 'haematomas' OR 'hematomas' OR 'hematoma s' OR 'hematomae' OR 'hemorrhaged' OR 'hemorrhagic' OR 'hemorrhagical' OR 'hemorrhaging') AND ('metabolite' OR 'metabolite s' OR 'metabolites') 9716  #10 ('milk' OR ('cow' AND 'milk') OR 'cow milk') AND ('economics' OR 'production' OR 'productions' OR 'efficiency' OR 'productivity' OR 'product' OR 'product s' OR 'productive' OR 'productively' OR 'productivities' OR 'products') 3000  #11 'meat' AND ('qualities' OR 'quality' OR 'quality s') 597  #12 #6 or #7 or #8 or #9 or #10 or #11 34878  #13 #5 and #12 6 |
| **Embase** | #13. #5 AND #12 57  #12. #6 OR #7 OR #8 OR #9 OR #10 OR #11 401,428  #11. 'meat':ti,ab,kw AND ('qualities':ti,ab,kw OR 13,170  'quality':ti,ab,kw OR 'quality s':ti,ab,kw)  #10. ('milk':ti,ab,kw OR ('cow':ti,ab,kw AND 'milk':ti,ab,kw) 47,133  OR 'cow milk':ti,ab,kw) OR 'human milk':ti,ab,kw OR  'milk':ti,ab,kw) AND ('economics':ti,ab,kw OR  'production':ti,ab,kw OR 'productions':ti,ab,kw  OR 'efficiency':ti,ab,kw OR  'productivity':ti,ab,kw OR 'product':ti,ab,kw OR  'product s':ti,ab,kw OR 'productive':ti,ab,kw OR  'productively':ti,ab,kw OR  'productivities':ti,ab,kw OR 'products':ti,ab,kw)  #9. ('blood':ti,ab,kw OR 'bloods':ti,ab,kw OR 55,840  'haematology':ti,ab,kw OR 'hematology':ti,ab,kw  OR 'haematoma':ti,ab,kw OR 'hematoma':ti,ab,kw OR  'haemorrhage':ti,ab,kw OR 'hemorrhage':ti,ab,kw  OR 'haemorrhages':ti,ab,kw OR  'hemorrhages':ti,ab,kw OR 'haemorrhagic':ti,ab,kw  OR 'haemorrhaging':ti,ab,kw OR  'hematologies':ti,ab,kw OR 'haematomas':ti,ab,kw  OR 'hematomas':ti,ab,kw OR 'hematoma s':ti,ab,kw  OR 'hematomae':ti,ab,kw OR 'hemorrhaged':ti,ab,kw  OR 'hemorrhagic':ti,ab,kw OR  'hemorrhagical':ti,ab,kw OR  'hemorrhaging':ti,ab,kw) AND  ('metabolite':ti,ab,kw OR 'metabolite s':ti,ab,kw  OR 'metabolites':ti,ab,kw)  #8. ('ruminal':ti,ab,kw OR 'ruminally':ti,ab,kw OR 4,110  'ruminant s':ti,ab,kw OR 'ruminants':ti,ab,kw OR  'ruminant':ti,ab,kw) AND ('ferment':ti,ab,kw OR  'fermentabilities':ti,ab,kw OR  'fermentability':ti,ab,kw OR  'fermentable':ti,ab,kw OR 'fermentate':ti,ab,kw  OR 'fermentated':ti,ab,kw OR  'fermentates':ti,ab,kw OR 'fermentation':ti,ab,kw  OR 'fermentations':ti,ab,kw OR  'fermentative':ti,ab,kw OR  'fermentatively':ti,ab,kw OR  'fermentator':ti,ab,kw OR 'fermented':ti,ab,kw OR  'fermenter':ti,ab,kw OR 'fermenters':ti,ab,kw OR  'fermenting':ti,ab,kw OR 'fermention':ti,ab,kw OR  'ferments':ti,ab,kw)  #7. 'carcass':ti,ab,kw OR 'carcasses':ti,ab,kw 20,977  #6. 'digest':ti,ab,kw OR 'digestability':ti,ab,kw OR 273,412  'digestable':ti,ab,kw OR 'digestate':ti,ab,kw OR  'digestates':ti,ab,kw OR 'digested':ti,ab,kw OR  'digester':ti,ab,kw OR 'digesters':ti,ab,kw OR  'digestibilities':ti,ab,kw OR  'digestibility':ti,ab,kw OR 'digestible':ti,ab,kw  OR 'digesting':ti,ab,kw OR 'digestion':ti,ab,kw  OR 'digestions':ti,ab,kw OR ('digestive':ti,ab,kw  AND 'system':ti,ab,kw) OR 'digestive  system':ti,ab,kw OR 'digestive':ti,ab,kw OR  'digestives':ti,ab,kw OR 'digests':ti,ab,kw  #5. #3 AND #4 150  #4. #1 OR #2 110,564  #3. 'oils, volatile':ti,ab,kw OR ('oils':ti,ab,kw AND 20,842  'volatile':ti,ab,kw) OR 'volatile oils':ti,ab,kw  OR ('essential':ti,ab,kw AND 'oils':ti,ab,kw) OR  'essential oils':ti,ab,kw  #2. 'cattle':ti,ab,kw OR ('dairy':ti,ab,kw AND 109,616  'cow':ti,ab,kw) OR 'dairy cow':ti,ab,kw  #1. 'calf':ti,ab,kw AND ('cattle':ti,ab,kw OR 5,299  'cow':ti,ab,kw) |
| Pubmed | 13 #5 and #12  12 #6 or #7 or #8 or #9 or #10 or #11  11 ("meat"[MeSH Terms] OR "meat"[All Fields]) AND ("qualities"[All Fields] OR "quality"[All Fields] OR "quality s"[All Fields])  10 ("milk"[MeSH Terms] OR "milk"[All Fields] OR ("cow"[All Fields] AND "milk"[All Fields]) OR "cow milk"[All Fields]) AND ("economics"[MeSH Terms] OR "economics"[All Fields] OR "production"[All Fields] OR "productions"[All Fields] OR "efficiency"[MeSH Terms] OR "efficiency"[All Fields] OR "productivity"[All Fields] OR "product"[All Fields] OR "product s"[All Fields] OR "productive"[All Fields] OR "productively"[All Fields] OR "productivities"[All Fields] OR "products"[All Fields])  9 ("blood"[MeSH Subheading] OR "blood"[All Fields] OR "blood"[MeSH Terms] OR "bloods"[All Fields] OR "haematology"[All Fields] OR "hematology"[MeSH Terms] OR "hematology"[All Fields] OR "haematoma"[All Fields] OR "hematoma"[MeSH Terms] OR "hematoma"[All Fields] OR "haemorrhage"[All Fields] OR "hemorrhage"[MeSH Terms] OR "hemorrhage"[All Fields] OR "haemorrhages"[All Fields] OR "hemorrhages"[All Fields] OR "haemorrhagic"[All Fields] OR "haemorrhaging"[All Fields] OR "hematologies"[All Fields] OR "haematomas"[All Fields] OR "hematomas"[All Fields] OR "hematoma s"[All Fields] OR "hematomae"[All Fields] OR "hemorrhaged"[All Fields] OR "hemorrhagic"[All Fields] OR "hemorrhagical"[All Fields] OR "hemorrhaging"[All Fields]) AND ("metabolite"[All Fields] OR "metabolite s"[All Fields] OR "metabolites"[All Fields])  8 ("ruminal"[All Fields] OR "ruminally"[All Fields] OR "ruminant s"[All Fields] OR "ruminants"[MeSH Terms] OR "ruminants"[All Fields] OR "ruminant"[All Fields]) AND ("ferment"[All Fields] OR "fermentabilities"[All Fields] OR "fermentability"[All Fields] OR "fermentable"[All Fields] OR "fermentate"[All Fields] OR "fermentated"[All Fields] OR "fermentates"[All Fields] OR "fermentation"[MeSH Terms] OR "fermentation"[All Fields] OR "fermentations"[All Fields] OR "fermentative"[All Fields] OR "fermentatively"[All Fields] OR "fermentator"[All Fields] OR "fermented"[All Fields] OR "fermenter"[All Fields] OR "fermenters"[All Fields] OR "fermenting"[All Fields] OR "fermention"[All Fields] OR "ferments"[All Fields])  7 "carcass"[All Fields] OR "carcasses"[All Fields]  6 "digest"[All Fields] OR "digestability"[All Fields] OR "digestable"[All Fields] OR "digestate"[All Fields] OR "digestates"[All Fields] OR "digested"[All Fields] OR "digester"[All Fields] OR "digesters"[All Fields] OR "digestibilities"[All Fields] OR "digestibility"[All Fields] OR "digestible"[All Fields] OR "digesting"[All Fields] OR "digestion"[MeSH Terms] OR "digestion"[All Fields] OR "digestions"[All Fields] OR "digestive system"[MeSH Terms] OR ("digestive"[All Fields] AND "system"[All Fields]) OR "digestive system"[All Fields] OR "digestive"[All Fields] OR "digestives"[All Fields] OR "digests"[All Fields]  5 #3 AND #4  4 #1 OR #2  3 "oils, volatile"[MeSH Terms] OR ("oils"[All Fields] AND "volatile"[All Fields]) OR "volatile oils"[All Fields] OR ("essential"[All Fields] AND "oils"[All Fields]) OR "essential oils"[All Fields]  2 "cattle"[MeSH Terms] OR "cattle"[All Fields] OR ("dairy"[All Fields] AND "cow"[All Fields]) OR "dairy cow"[All Fields]  1 "calf"[All Fields] AND ("cattle"[MeSH Terms] OR "cattle"[All Fields] OR "cow"[All Fields]) |
| Web of Science | #13: #5 and #12 Results: 934  #12: #6 or #7 or #8 or #9 or #10 or #11 Results: 3029810  #11: TS="meat" AND (TS="qualities" OR TS="quality" OR TS="quality s") Results: 82658  #10: (TS="milk" OR (TS="cow" AND TS="milk")) AND (TS="economics" OR TS="production" OR TS="productions" OR TS="efficiency" OR TS="productivity" OR TS="product" OR TS="product s" OR TS="productive" OR TS="productively" OR TS="productivities" OR TS="products" ) Results: 193807  #9: (TS="blood" OR TS="bloods" OR TS="haematology" OR TS="hematology" OR TS="haematoma" OR TS="hematoma" OR TS="haemorrhage" OR TS="hemorrhage" OR TS="haemorrhages" OR TS="hemorrhages" OR TS="haemorrhagic" OR TS="haemorrhaging" OR TS="hematologies" OR TS="haematomas" OR TS="hematomas" OR TS="hematoma s" OR TS="hematomae" OR TS="hemorrhaged" OR TS="hemorrhagic" OR TS="hemorrhagical" OR TS="hemorrhaging" ) AND (TS="metabolite" OR TS="metabolite s" OR TS="metabolites" ) Results: 150748  #8: (TS="ruminal" OR TS="ruminally" OR TS="ruminant s" OR TS="ruminants" OR TS="ruminant") AND (TS="ferment" OR TS="fermentabilities" OR TS="fermentability" OR TS="fermentable" OR TS="fermentate" OR TS="fermentated" OR TS="fermentates" OR TS="fermentation" OR TS="fermentations" OR TS="fermentative" OR TS="fermentatively" OR TS="fermentator" OR TS="fermented" OR TS="fermenter" OR TS="fermenters" OR TS="fermenting" OR TS="fermention" OR TS="ferments") Results: 19660  #7: TS="carcass" OR TS="carcasses" Results: 58002  #6: TS="digest" OR TS="digestability" OR TS="digestable" OR TS="digestate" OR TS="digestates" OR TS="digested" OR TS="digester" OR TS="digesters" OR TS="digestibilities" OR TS="digestibility" OR TS="digestible" OR TS="digesting" OR TS="digestion" OR TS="digestions" OR TS="digestive system" OR (TS="digestive" AND TS="system") OR TS="digestive system" OR TS="digestive" OR TS="digestives" OR TS="digests" Results: 2661125  #5: #3 AND #4 Results: 1462  #4: #1 OR #2 Results: 546525  #3: TS="oils, volatile" OR (TS="oils" AND TS="volatile") OR TS="volatile oils" OR (TS="essential" AND TS="oils") OR TS="essential oils" Results: 72693  #2: TS="cattle" OR (TS="dairy" AND TS="cow") OR TS="dairy cow" Results: 545908  #1 TS="calf" AND (TS="cattle" OR TS="cow") Results: 41557 |
